# Kaposi Sarcoma as Presentation of HIV – A Clinical Case

**DOI:** 10.7759/cureus.18936

**Published:** 2021-10-21

**Authors:** Rita Costa, Leonor Silva, Renata Monteiro, Filipa Santos, Margarida Mota

**Affiliations:** 1 Internal Medicine, Centro Hospitalar de Vila Nova de Gaia/Espinho, Vila Nova de Gaia, PRT

**Keywords:** opportunistic infections, cardiomyopathy, paclitaxel, haart, hiv, kaposi sarcoma

## Abstract

Kaposi sarcoma (KS) is the most common neoplasm of people with human immunodeficiency virus (HIV) infection. Although, in the antiretroviral therapy (ART) era, KS is a rare form of presentation of HIV/acquired immunodeficiency syndrome. The authors present a case of disseminated KS in a 23-year-old male. Just after the diagnosis the patient started ART and then chemotherapy with placlitaxel with clinical improvement. This case is highly representative of the complexity of HIV. The authors aim to bring awareness of an unusual form of presentation of HIV, and recall the severity and the necessity of an early diagnosis and treatment.

## Introduction

Kaposi sarcoma (KS) is the most common neoplasm of patients with human immunodeficiency virus (HIV) infection [1]. In the antiretrovirals era, the incidence of KS decreased from 15.2/1000 patient-year to 4.9/1000 patient-year and so; nowadays, KS is an unusual presentation of HIV [2]. KS is a multifocal angioproliferative neoplasm associated with the infection by human herpes virus type 8 (KSHV) [1]. KSHV has been shown to be the etiologic agent for several other tumors and diseases, including primary effusion lymphoma (PEL), an extracavitary variant of PEL, KS-associated diffuse large B-cell lymphoma, a form of multicentric Castleman disease (MCD), and KS inflammatory cytokine syndrome (KICS). KICS is an entity recently described in patients with HIV and KSHV. The syndrome is characterized by lymphadenopathy, pancytopenia, and signs of systemic inflammatory syndrome [3]. Lymph node, bone marrow, or splenic biopsy can be used to distinguish from MCD [3]. KICS has a higher mortality than MCD [3].

Cutaneous KS presents as red, violaceous (purple), or brown lesions, from macules, patches, and papules to nodules or tumors. These lesions of the skin are highly characteristic, facilitating the diagnosis, although disseminated disease may affect any organ. The most common sites of disease dissemination include the skin, mucosal surfaces, respiratory tract, and lymph nodes, and extensive disseminated disease is often associated with lymphedema [4].

KS makes a differential diagnosis with bacillary angiomatosis, nevus, and B-cell lymphoma. Biopsy is the gold standard for diagnosis [5].

## Case presentation

A 23-year-old male with type I diabetes resorted to an outpatient clinic complaining of disseminated dermatosis. The patient referred multiple non-pruritic skin lesions over his chest with one-year evolution, with further progression to his arms and limbs. He mentioned a significant involuntary weight loss of 15%, associated with fever predominantly in the afternoon for the last two months. He also experienced diarrhea persisting for more than two weeks. Anorexia and night sweats were denied.

He had an unprotected heterosexual exposure in the past. There was no history of blood transfusion, injection drug use, or needle sharing. His only medication was insulin. 

**Figure 1:**
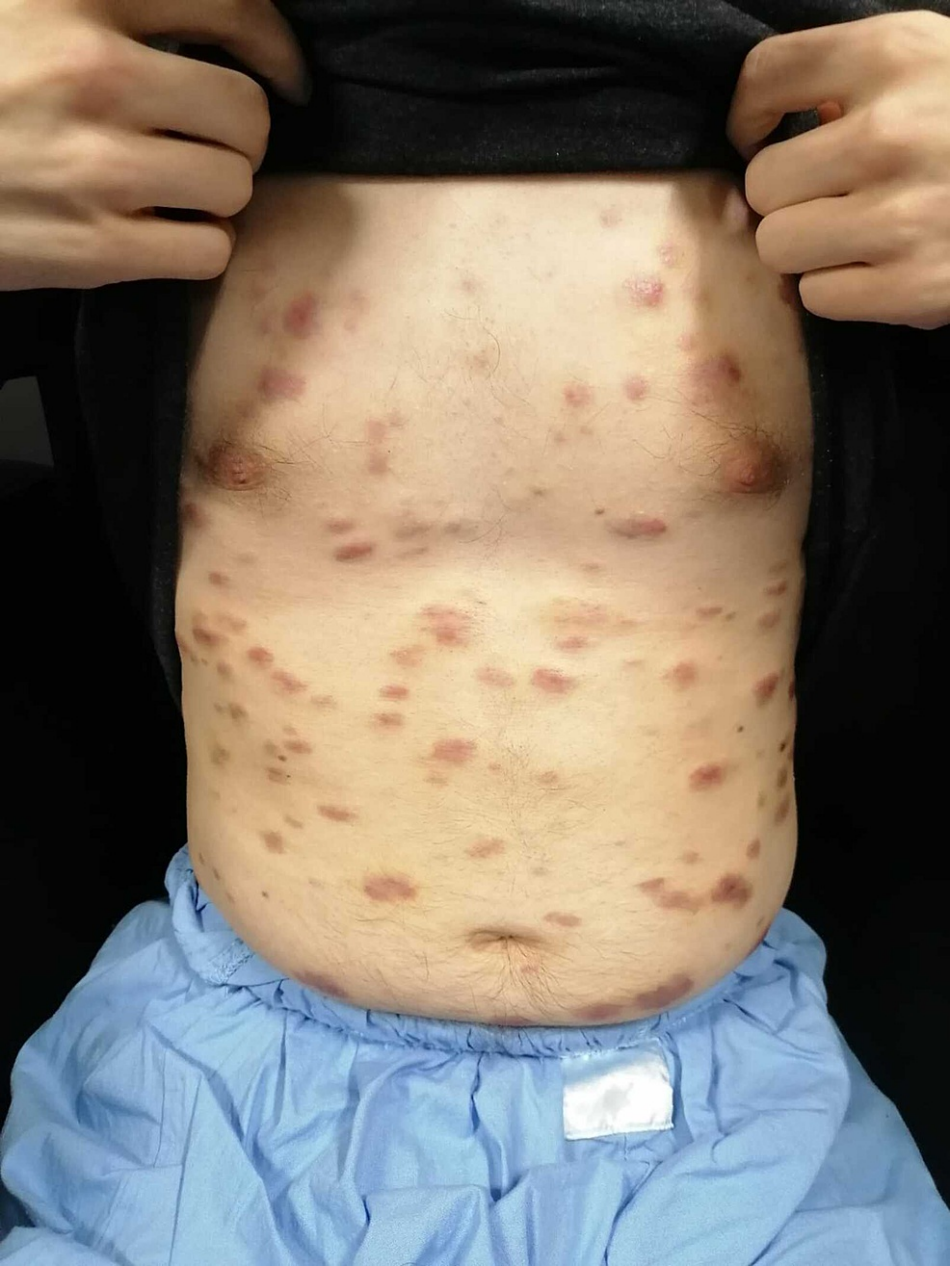
Violaceous papules

On physical examination, the patient was alert, oriented but emaciated, with multiple violaceous papules and nodules in his trunk, arms, and legs (Figure 1). Oral mucosa was not affected. He had no palpable lymphadenopathy nor splenomegaly. His vitals showed normal blood pressure of 120/72 mmHg, sinus tachycardia around 140-150 pulse per minute, and temperature of 36.5ºC. On lung examination, he presented with diminished breath sounds bilaterally with no other alterations in the physical examination. Oxygen saturation on room air was 97%.

**Figure 2:**
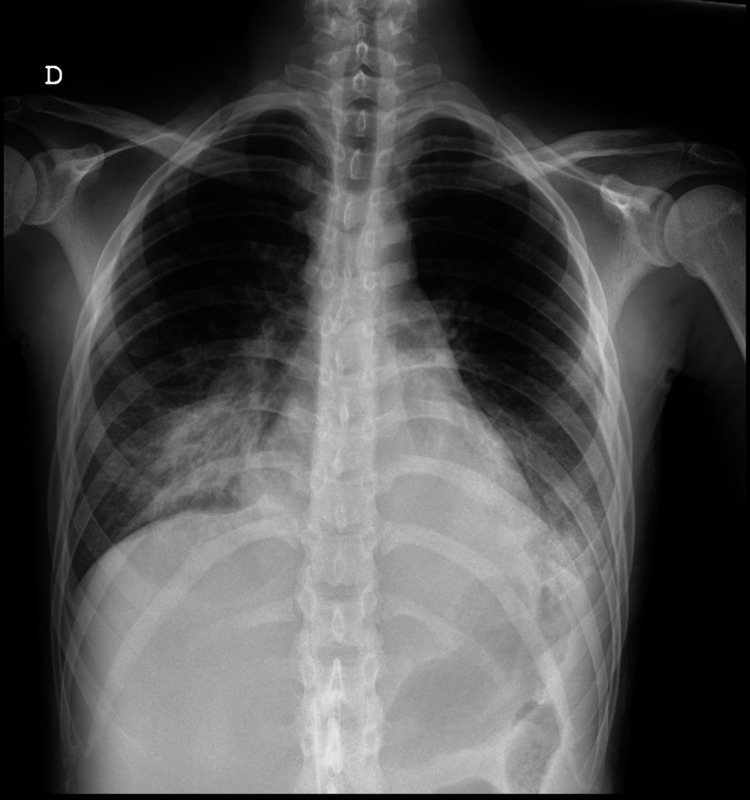
Chest x-ray with hypotransparency of the right lower lobe

The lab results showed hemoglobin 9.4 g/dL, white blood cells 2,770/uL, lymphocytopenia 810/uL, CD4+ T lymphocyte count of 23 cells/mm^3^, normal renal function, no cytocolestase (total bilirubin 0.27 mg/dL, glutamic-oxaloacetic transaminase 23 U/L, pyruvic transaminase 13 U/L, gamma-glutamyl transferase 35 U/L, and alkaline phosphatase 89 U/L), C-reactive protein 1.92 mg/dL, and sedimentation velocity 67 mm/h. HIV1 serology (fourth-generation test) was positive, and the HIV viral load (VL) of 1,820,000 copies/mm^3^. Chest x-ray demonstrated an hypotransparency of the right lower lobe (Figure 2).

**Figure 3:**
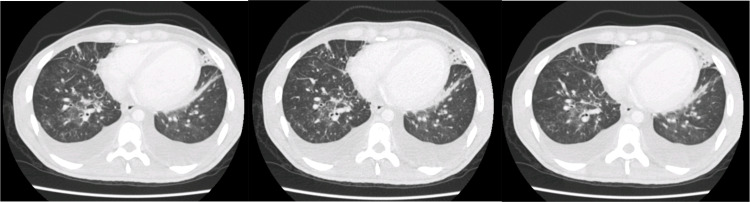
Thorax CT shows bilateral pleural effusion and nodular areas, with a ground glass pattern, associated with septal interlobular thickening CT, computed tomography

A chest computed tomography (CT) showed bilateral pleural effusion and vaguely nodular areas with ground glass pattern, more evident in the right lower lobe, associated with thickening of the interlobular septa (Figure 3). These lesions, considering the context, were suggestive of pulmonary KS, although lymphoma, tuberculosis, fungal infection, or other opportunistic infections could not be excluded.

Skin biopsy of the lesion demonstrated dermal vascular proliferation with thin-walled and irregular vessels. Endothelial cells formed a disorganized monolayer with dissolution of collagen fibers and perianexial infiltration. Immunohistochemistry was positive for CD34, ERG, and KSHV. These findings are compatible with KS skin lesions.

Bronchoscopy and bronchoalveolar lavage (BAL) were performed. Direct examination with gram stain and cultural examination BAL were negative for the presence of bacteria. Also, cultural mycological examination and direct examination, molecular biology, and cultural examination for mycobacterium were negative. The BAL cytology excluded the presence of malignant cells. *Pneumocystis jiroveci* was also excluded by polymerase chain reaction. Although the characteristic lesions, as the violaceous lesions, were not identified on bronchoscopy and the BAL was negative for malignant cells, given the changes in the chest CT, the most likely diagnosis was KS with pulmonary involvement.

Stool cultures were negative for parasites (*Giardia and Cryptosporidium*), bacterial (cultural examination performed for *Salmonella *spp*, Shigella *spp*, Yersinia *spp*, Campylobacter *spp*, Escherichia coli *O157:H7, and *vibrionaceas*), and *Clostridiodes difficile *(glutamate dehydrogenase antigen and toxin A and B screening). Colonoscopy exhibited two small ulcers on transverse colon, while the biopsy revealed colorectal mucosa with colitis, positive for cytomegalovirus (CMV) (histological examination suggestive of viral inclusions by CMV, confirmed by immunohistochemistry). Histology was negative for KS and no expression of KSHV was observed.

An ophthalmologic evaluation revealed perivascular retinal opacification in the infero-nasal quadrant of the right eye suggestive of CMV retinitis.

The patient completed 21 days of treatment with valganciclovir (900 mg twice daily) with resolution of gastrointestinal complaints followed by secondary prophylaxis valganciclovir (900 mg once daily).

Soon after the diagnosis the patient started antiretroviral therapy (ART) and was proposed to chemotherapy. With that aim, he underwent a transthoracic echocardiogram that exhibited moderate mitral regurgitation and severe depression of left ventricular systolic function, with a mean ejection fraction of 21%. He underwent Holter monitoring, which also revealed sinus tachycardia (average 118 beats per minute). Cardiac magnetic resonance imaging (MRI) showed dilated cardiomyopathy (non-ischemic etiology) with severe impairment of biventricular systolic function, and intramural fibrosis at the level of the basal and middle segments of the interventricular septum - stria mesocardial. The fibrosis pattern described can be found in the context of post-myocarditis, familial or idiopathic etiology, and associated with poor prognosis. Although the patient had no family history of sudden cardiac death, syncope, or left ventricular hypertrophy, genetic testing for cardiomyopathies was performed and confirmed the suspicion of hereditary cardiomyopathy (the c.43009del p. variant of the TNN gene, in heterozygosity; and the c.4046G>A variant in the DSP gene, of uncertain significance). He was initiated on prognostic modifying therapy for heart failure (as angiotensin receptor-neprilysin inhibitor, beta blocker, and aldosterone receptor antagonist).

At this point, it is not clear whether the heart failure is explained by the HIV involvement of the heart or the presence of hereditary cardiomyopathy. The echocardiogram was repeated after four months and revealed a great improvement, already with normal left ventricular systolic function.

The patient was treated with paclitaxel (100 mg/m^2^, every 15 days), and after six cycles he presented with partial response with a great improvement of the lung lesions and was kept in the same chemotherapy scheme. Five months after ART (darunavir 800 mg plus cobicistat 150 mg, emtricitabine 200 mg, and tenofovir alafenamide) and chemotherapy the patient still has countless violaceous lesions on the trunk and upper limbs, now more tenuous and without relief. The patient has HIV VL of 45 copies/mL and 38 CD4+ T cells.

## Discussion

The clinical manifestations in our patient highlight the potentially aggressive course of KS in people living with HIV (PLWH). The disseminated presentation of acquired immunodeficiency syndrome (AIDS)-associated KS has a poor prognosis [3]. Pulmonary involvement generally occurs in severely immunosuppressed patients who already have mucocutaneous or digestive involvement, though 15% of patients with pulmonary KS have no mucocutaneous lesions at diagnosis [6].

Pulmonary involvement is particularly important, as it is associated with worse prognosis and increased mortality compared with other systems [7]. In a cohort of 305 HIV-1-infected patients diagnosed with KS since 1996, the median survival time for patients with pulmonary KS was 19 months compared with a median survival time of four months reported for the same cohort in the pre-ART era [8].

Given the frequency of opportunistic pulmonary infections in PLWH, diagnosing lung involvement is often a challenge. Even bronchoscopy may fail to establish the diagnosis. Mitchell and Miller reported that only 45% of cases have endobronchial lesions located at segmental orifices in the main trachea or bronchi that are reachable with a bronchoscope [9]. In pre-ART era, the estimated incidence of pulmonary KS in patients living with AIDS was around 30% [10].

According to AIDS Clinical Trials Group system for AIDS-related KS, patients are assessed on the extent of tumor (T), the status of the immune system (I), and the presence of systemic illness (S). In the era of ART, CD4 level does not seem to provide prognostic information. Two different risk categories were identified: a good risk (T0S0, T1S0, T0S1) and a poor risk (T1S1). Our patient presented with high-risk factors: widespread KS lesions (T1) and systemic illness (opportunistic infection and B symptoms - as fever, night sweats, and significant weight loss).

Currently, there is no cure for KS; therefore, the aim of the therapy is to slow disease progression and alleviate symptoms. Therapeutic options depend on the extent and rate of tumor growth, VL, and CD4+ T lymphocyte count. Localized skin disease can be treated with ART and some local therapies (laser CO_2_, cryotherapy, radiotherapy, intralesional vinblastine) [11]. There are no defined criteria for systemic KS therapy, and the decision should be individualized. Systemic KS therapy is usually administered to patients with widespread T1 disease, extensive cutaneous KS, symptomatic or life-threatening visceral KS, ulcerating KS, KS associated with edema, or tumor-related pain [12]. Also, systemic therapy is justified in patients that fail to respond to ART [12]. A 2014 review suggested that ART plus chemotherapy may be beneficial in reducing disease progression compared to ART alone in patients with severe or progressive KS [13]. Cytotoxic chemotherapy represents the standard of care, and liposomal formulations of doxorubicin or daunorubicin and paclitaxel show similar clinical efficacy [13,14]. However, KSHV cannot be eradicated; tumors may recur and patients often require additional therapies. Chronic administration of cytotoxic agents is poorly tolerated, and in this setting, drugs such as pomalidomide/lenalidomide may be discussed [11].

Importantly, patients co-infected with KS and HIV often develop more than one KSHV-associated disease. Active KSHV replication has also been associated with KICS. The clinical presentation mimics sepsis with respiratory failure and often leading to mechanical ventilation and vasopressor use. However, these patients do not improve with standard antibiotic therapy. It occurs due to overproduction of KSHV-related interleukins (IL-6 and IL-10) and symptoms are from the associated cytokine storm. Although there is no established gold standard of treatment, addition of immunomodulatory therapy such as rituximab to the ongoing ART has been shown to result in improvement in clinical status. KICS has a high mortality rate if left untreated and early recognition and management is important to improve patient outcomes. It has been hypothesized that treating the underlying tumor may decrease KSHV-associated cytokines. However, in situations where this syndrome is present, treating the original sarcoma is not easy due to related comorbidities. Another drug that may be used is ganciclovir, which has activity against KSHV. Finally, liposomal doxorubicin can be used to eliminate KS spindle cells and prevent the aggressive proliferation of KS lesions with concurrent rituximab treatment [15].

## Conclusions

The risk of AIDS-related KS has declined since the introduction of ART in the mid-1990s. However, it remains highly prevalent in PLWH and can arise at any time during the course of HIV infection. It accelerates the clinical course of HIV infection, and generally occurs at CD4 count <200 cells/mm^3^. Response to treatment is variable according to lesion extension and patient immunity status. 

It is now appreciated that KSHV can cause several diseases, several of which had not been previously recognized.

This case emphasizes the need to recognize these skin lesions, and strongly consider pulmonary KS as a possible cause for respiratory illness in any PLWH with low CD4 counts. Chest CT findings can play an important role in the diagnosis of pulmonary KS, since characteristic patterns may be observed. BAL must be performed in all pulmonary KS patients to rule out the possibility of concomitant infection.

This case reflects the complexity of HIV and reinforces the need for greater awareness in screening regardless of whether a clear risk behavior has been identified.
